# Transcriptome Characterization of *Gnetum parvifolium* Reveals Candidate Genes Involved in Important Secondary Metabolic Pathways of Flavonoids and Stilbenoids

**DOI:** 10.3389/fpls.2016.00174

**Published:** 2016-03-04

**Authors:** Nan Deng, Ermei Chang, Minghe Li, Jing Ji, Xiamei Yao, Igor V. Bartish, Jianfeng Liu, Jing Ma, Lanzhen Chen, Zeping Jiang, Shengqing Shi

**Affiliations:** ^1^State Key Laboratory of Tree Genetics and Breeding, Research Institute of Forestry, Chinese Academy of ForestryBeijing, China; ^2^College of Landscape Architecture, Fujian Agriculture and Forestry UniversityFuzhou, China; ^3^Department of Genetic Ecology, Institute of Botany, Academy of Sciences of the Czech RepublicPraha, Czech Republic; ^4^Institute of Apicultural Research, Chinese Academy of Agricultural SciencesBeijing, China; ^5^Risk Assessment Laboratory for Bee Products, Quality and Safety of Ministry of AgricultureBeijing, China

**Keywords:** Gnetophyta, *Gnetum*, evolution, natural bioactive compounds, gene expression

## Abstract

*Gnetum* is a small, unique group of Gnetophyta with a controversial phylogenetic position. *Gnetum parvifolium* is an important Chinese traditional medicinal plant, which is rich in bioactive compounds such as flavonoids and stilbenoids. These compounds provide significant medicinal effects, mostly as antioxidant, anticancer, and antibacterial agents. However, the mechanisms involved in the biosynthesis and regulation of these compounds in *G. parvifolium* are still unknown. In this study, we found that flavonoids and stilbene compounds accumulated at different levels in various tissues of *G. parvifolium*. We further obtained and analyzed massive sequence information from pooled samples of *G. parvifolium* by transcriptome sequencing, which generated 94,816 unigenes with an average length of 724 bp. Functional annotation of all these unigenes revealed that many of them were associated with several important secondary metabolism pathways including flavonoids and stilbenoids. In particular, several candidate unigenes (*PAL-, C4H-, 4CL-*, and *STS-like* genes) involved in stilbenoids biosynthesis were highly expressed in leaves and mature fruits. Furthermore, high temperature and UV-C strongly induced the expression of these genes and enhanced stilbene production (i.e., resveratrol and piceatannol) in leaves of young seedlings. Our present transcriptomic and biochemical data on secondary metabolites in *G. parvifolium* should encourage further investigation on evolution, ecology, functional genomics, and breeding of this plant with strong pharmaceutical potential.

## Introduction

*Gnetum* (35–40 species), together with two other genera (*Ephedra* and *Welwitschia*), comprise a small and unique group of Gnetophyta, whose phylogenetic position within the seed plants (Spermatophyta) is controversial (Zhong et al., 2010; Shi S. Q. et al., 2011). However, it might provide important insights into the evolution and the origin of flowers (Crane et al., 1995; Wu et al., 2007; Zhong et al., 2010). In addition to their striking evolutionary divergence, many species of *Gnetum* are rich sources of raw materials for traditional medicines, and they are widely used to relieve swelling, treat acute respiratory infections, and cure chronic bronchitis (Wang and Liang, 2006). These plants are also rich in diverse natural bioactive compounds, such as flavonoids and stilbenoids, identified by spectrophotometry, nuclear magnetic resonance, and X-ray crystallographic analyses (Lin et al., 1991, 1992; Deng et al., 2014). These metabolites have hypotensive, antioxidant, anticancer, and antibacterial effects (Fang et al., 2012, 2013; Kongkachuichai et al., 2015). Furthermore, some *Gnetum* species, such as *G. africanum* and *G. gnemon*, have been used widely as healthy vegetables and fruits in southeast Asia and Central Africa (Ali et al., 2011; Bhat and binti Yahya, 2014; Kongkachuichai et al., 2015).

Many studies show that *Gnetum* is rich in flavonoids (Lan et al., 2013, 2014; Bhat and binti Yahya, 2014; Deng et al., 2014). However, only a few of them have been identified, such as chrysoeriol (5,7,4′-trihydroxy-3′-methoxyflavone) from *G. montanum* (Xiang et al., 2002; Saisin et al., 2009) and 5,7,2′-trihydroxy-5′-methoxyflavone from *G. macrostachyum* (Saisin et al., 2009). These compounds show radical scavenging activity against 1,1-diphenyl-2-picrylhydrazyl (Saisin et al., 2009). In addition, stilbenoids, a family of polyphenols well known for their diverse biological activities, have been found in at least 15 *Gnetum* species. Together with plants from the Cyperaceae, Dipterocarpaceae, Fabaceae, Pinaceae, and Vitaceae, this genus is considered as one of the top sources of stilbenoids, which have a limited, but heterogeneous, distribution within the plant kingdom (Riviere et al., 2012). Around 100 different types of stilbenoids, representing almost the full spectrum of natural stilbenoids known to date, have been found in *Gnetum* (Wang and Liang, 2006; Shi S. Q. et al., 2011; Riviere et al., 2012). Stilbenoids include monomers (such as resveratrol, oxyresveratrol, isorhapontigenin, and piceatannol), oligomers (formed from the heterogeneous oligomerization of several monomers), stilbene glucosides (Iliya et al., 2006), and some other derivatives of stilbenes (conjugated with flavanols or lignans) (Riviere et al., 2012).

To date, natural products of flavonoids and stilbenoids have attracted much attention, not only because they play an important role in plants' response to stress conditions (Di et al., 2012), but also since they act as potential targets for the pharmaceutical and nutraceutical industries (Katsuyama et al., 2007). Uncovering the health benefits associated with these bioactive compounds has resulted in an explosion of research on their medicinal properties, particularly focused on the stilbene compound, resveratrol (Watts et al., 2006). One of the most exciting findings is that some stilbenes and their derivatives show potent inhibitory activities against cancer (Fang et al., 2013). For example, isorhapontigenin, a new derivative of stilbene from *G. cleistostachyum*, has been identified as a major anti-cancer compound, acting via down-regulation of an X-linked inhibitor of apoptosis protein (Fang et al., 2013). Derivatives of resveratrol from *G. gnemon* can suppress multiple angiogenesis-related endothelial cell functions and/or tumor angiogenesis (Kunimasa et al., 2011). Resveratrol, isorhapontigenin, pinosylvin, and other stilbene compounds isolated from *Gnetum parvifolium* display significant inhibition of HIV-1 replication, and potent inhibitory activity in the Maillard reaction (Tanaka et al., 2001; Piao et al., 2010). These studies of natural oligostilbenes from *Gnetum* attract an increasing attention due to their health effects on humans in recent years.

Flavonoids and stilbenes are synthesized by a common pathway, with chalcone synthases (CHSs) and stilbene synthases (STSs) as key branch enzymes, respectively (Watts et al., 2006; Katsuyama et al., 2007). STSs have likely developed from CHSs during evolution (Tropf et al., 1994). Both enzymes use the same substrate, *p*-coumaroyl-CoA, generated from the phenylpropanoid pathway undergoing the initial three steps of the pathway catalyzed by phenylalanine ammonia-lyase (PAL), cinnamate 4-hydroxylase (C4H), and 4-coumaroyl CoA-ligase (4CL; Vogt, 2010). Both stilbene and chalcone ring structures can be produced in this pathway (Watts et al., 2006). However, genes encoding the enzymes involved in the biosynthesis of these bioactive compounds have not yet been characterized in *Gnetum*. The development of high-throughput sequencing technologies makes it possible to explore functional genomics in *Gnetum*. The subsequent identification of potential candidate genes, involved in the biosynthetic pathways of flavonoids and stilbenoids, would provide a better understanding on the biosynthesis and genetic regulation of these bioactive compounds in *Gnetum*.

Our previous studies have shown that *G. parvifolium* has high contents of total flavones, resveratrol, isorhapontigenin, and gnetol (Lan et al., 2013, 2014). Here, we obtained transcriptome data from a pooled RNA sample of young seedlings (roots, stems, and leaves) and mature trees (roots, stems, leaves, flowers, fruit flesh, and seeds) of *G. parvifolium* using RNA-seq approach, in combination with gene expression profiles and metabolite profiles in normal conditions and under stresses. We aimed to decipher the biosynthetic pathways of important secondary metabolites, including flavonoids and stilbenoids, which would pave the way for understanding and potentially *in vitro* synthesizing or engineering of these bioactive compounds in other medicinal plants. This study can also provide valuable information for breeding of populations of *Gnetum* that are rich in these bioactive compounds for human health.

## Materials and methods

### Sample collection and stress treatments

Collection of different tissues of *G. parvifolium* included seeds (five stages from inflorescence to mature seed, including fruits), germinated seeds (four stages based on the size of the embryo), and young inflorescences, together with leaves, roots, stems, shoot apices from both mature trees and young seedlings.

Treatments of short wavelength ultraviolet (UV-C) and high temperature: 1-year-old *G. parvifolium* seedlings cultivated in the greenhouse were transferred to a growth chamber for several-day acclimation, and then divided into two groups: one group was exposed to UV-C irradiation (20 W; the wavelength range was 200–275 nm) and the other was exposed to high temperature (40°C), for 0, 3, 6, 12, 24, and 48 h. Each treatment was repeated with four biological replicates.

The leaves were collected at the designated stress time points, immediately frozen in liquid nitrogen, and then stored at −80°C for RNA isolation and measurements of secondary metabolites.

### RNA isolation

Total RNA was isolated from different samples (about 100 mg) according to the instruction of TRizol (Invitrogen, CA, USA). The purity of RNA was checked using a NanoPhotometer® spectrophotometer (Implen, CA, USA). The concentrations were measured using a Qubit® RNA Assay Kit in a Qubit® 2.0 Fluorometer (Life Technologies, CA, USA). RNA integrity was assessed using a Nano 6000 Assay Kit for the Agilent Bioanalyzer 2100 system (Agilent Technologies, CA, USA).

### Construction of cDNA library and transcriptome sequencing

Three micrograms of pooled RNA from all the designated tissues (two biological replicates) were used as input material for transcriptome sequencing. The cDNA libraries were generated from purified mRNA using a NEBNext® Ultra™ RNA Library Prep Kit for Illumina® (NEB, MA, USA) following the manufacturer's recommendations, and index codes were added to attribute sequences to each sample. The library was sequenced on an Illumina Hiseq 2000 platform in Novogene (Beijing, China), which generated paired-end reads.

### Quality control

Raw data (raw reads) of fastq format was firstly processed through in-house perl scripts. In this step, clean data (clean reads) was obtained by removing reads containing adapter, reads containing poly-N and low quality reads with more than 10% Q < 20 bases [Q = −10log_10_(e), which indicates the base quality; e indicates the sequencing error rate] from raw data. Meanwhile, Q20, Q30, and GC content of the clean data were calculated. Only clean sequences with high quality were used for further analysis.

### Transcriptome assembly

*De novo* transcriptome assembly of the clean reads was performed using the Trinity software (Grabherr et al., 2011) with the parameter of min_kmer_cov set to 2 as default and all other parameters were also set as default. The expression level of each assembled transcript was measured using the fragments per kilobase per million mapped reads (FPKM) values (Mortazavi et al., 2008). All fragments were mapped onto the non-redundant set of transcripts to quantify the abundance of the assembled transcripts. The optimal assembly sequences were chosen as unigenes according to the assembly evaluation and length.

### Functional annotation

The unigenes were compared against the databases of Nr, Nt, and Swiss-Prot with *e*-value < 10E-5, and database of PFAM with *e*-value < 10E-2. Gene names were assigned to each assembled sequence based on the best BLAST hit (highest score). The BLAST results were initially imported into Blast2GO (Conesa et al., 2005) to annotate the unigenes with Gene Ontology (GO) terms with *e*-value < 10E-6, and then their functions were further predicted and classified by analysis against the Clusters of orthologous eukaryotic genes (KOG) database with *e*-value < 10E-3. Kyoto Encyclopedia of Genes and Genomes (KEGG) pathways (*e*-value < 10E-10) were assigned to the unigenes using the online KEGG Automatic Annotation Server (KAAS). The bi-directional best hit method was used to obtain KEGG Orthology (KO) assignments (Moriya et al., 2007). We used the indicated thresholds, which might be considered in general as not so rigorous, to get a wider source of sequence information for our analyses. We predicted that it might be possible to obtain additional genetic information by including some conserved domains even if the identified by our search unigenes have low hit lengths. The extra genetic information might be useful for the researchers who are interested in a detailed trancriptomic overview of *Gnetum*.

### Identification of simple sequence repeats (SSRs)

Assembled unigenes were used to detect SSRs by the microsatellite identification tool MISA (Version 1.0, Dec. 01, 2014; http://pgrc.ipk-gatersleben.de/misa/misa.html). Repeats of dinucleotides (>6), tri-, tetra-, penta-, and hexanucleotide repeats (>5) were considered as search criteria in the MISA script. But repeats of mononucleotides were excluded considering that the Illumina technology can make base call errors at long homopolymer stretches, which can result in misidentification of some SSRs (Quail et al., 2012).

### Determination of gene expression levels by qRT-PCR

Equal amounts of total RNA (1.0 μg) from the corresponding tissues (leaves, stems, roots from young seedlings and mature trees; fruit flesh and seeds) were reverse-transcribed by Superscript III Reverse Transcriptase (Invitrogen). The PCRs were performed according to the instructions of the SYBR premix Ex Taq™ kit (Takara, Dalian, China) and using a Roche LightCycler® 480 (Roche, IN, USA). Gene-specific primers were designed using Primer3 (v. 0.4.0, Nov. 20, 2012; http://frodo.wi.mit.edu/primer3/; Rozen and Skaletsky, 2000). The reaction was performed in a 20 μL volume, containing 10 μL of 2 × SYBR Green Mastermix (Takara), 300 nM of each primer and 2 μL of 10-fold diluted cDNA template. The PCR reactions were run in a Bio-Rad Sequence Detection System using the following program: 95°C for 10 s, and 40 cycles of 95°C for 15 s and annealing at 60°C for 30 s. Their relative expression levels were calculated via the 2^−ΔΔCt^ method (Ct, cycle threshold; Vandesompele et al., 2002).

### Extraction and quantification of flavonoids and stilbenoids

#### Extraction

The samples from different tissues or treatments were dried in the oven, and then ground into powders. The equal amounts of sample powders (10 mg) were immersed in methanol solution (80%, 500 μL), and processed with the aid of ultrasonic treatment for 30 min followed by an incubation at 4°C overnight. The homogenates were centrifuged at 12 000 rpm for 10 min and the supernatant was collected and stored at 4°C for further analysis.

#### Total flavonoids

According to NaNO_2_-Al(NO_3_)_3_-NaOH spectrophotometric method, 50 μL of the extract was transferred into 1 mL tube with 450 μL ddH_2_O, then 30 μL NaNO_2_ was added before shaking, and the reaction mixture was left for 5 min. Then, 30 μL of 10% Al(NO_3_)_3_ solution was added to the tube, mixed, and left to stand for 10 min at room temperature. After this, 200 μL of 1 mol/L NaOH solution was added to the tube, followed by the addition of ddH_2_O up to a volume of 1 mL. The absorbance of the mixtures was measured at 510 nm, and contents of the total flavonoids were calculated with quercetin (Tongtian Biotech. Co., Shanghai, China) as standard.

#### Total stilbenoids

Fifty microliters of the extract were diluted in ddH_2_O to a volume of 500 μL and measured at a wavelength of 333 nm. Eighty percent methanol was used as reference and resveratrol (Tongtian Biothech) was used as standard for quantification.

#### Analysis by HPLC

According to the method of Jiang et al. (2015), 20 μL of the extract was run on an HPLC 1260 (Agilent, CA, USA) system with an Eclipse XDB-C18 reverse phase column (4.6 × 150 mm, particle size 5 μm). Compounds were separated with a linear eluting gradient (5–70% solvent B over 30 min) with solvent A (0.1% formic acid in water) and solvent B (0.1% formic acid in acetonitrile) at flow rate of 1 mL·min^−1^. A photodiode array detector (Agilent) was used for the detection of UV-visible absorption from 190 to 600 nm. The chemical standards included flavonoids (anthocyanins, apigenin, genistein, isoflavoues, aglycone, isorhamnetin, kaempferol, luteolin, morin hydrate, quercetin, rutinum, and tricin), and stilbenoids (ε-viniferin, isorhapontigenin, rhapontigenin, resveratrol, piceatannol, pinosylvin, and gnetol).

## Results

### Quantification of total flavonoids and stilbenes in different tissues

**Figure 1:**
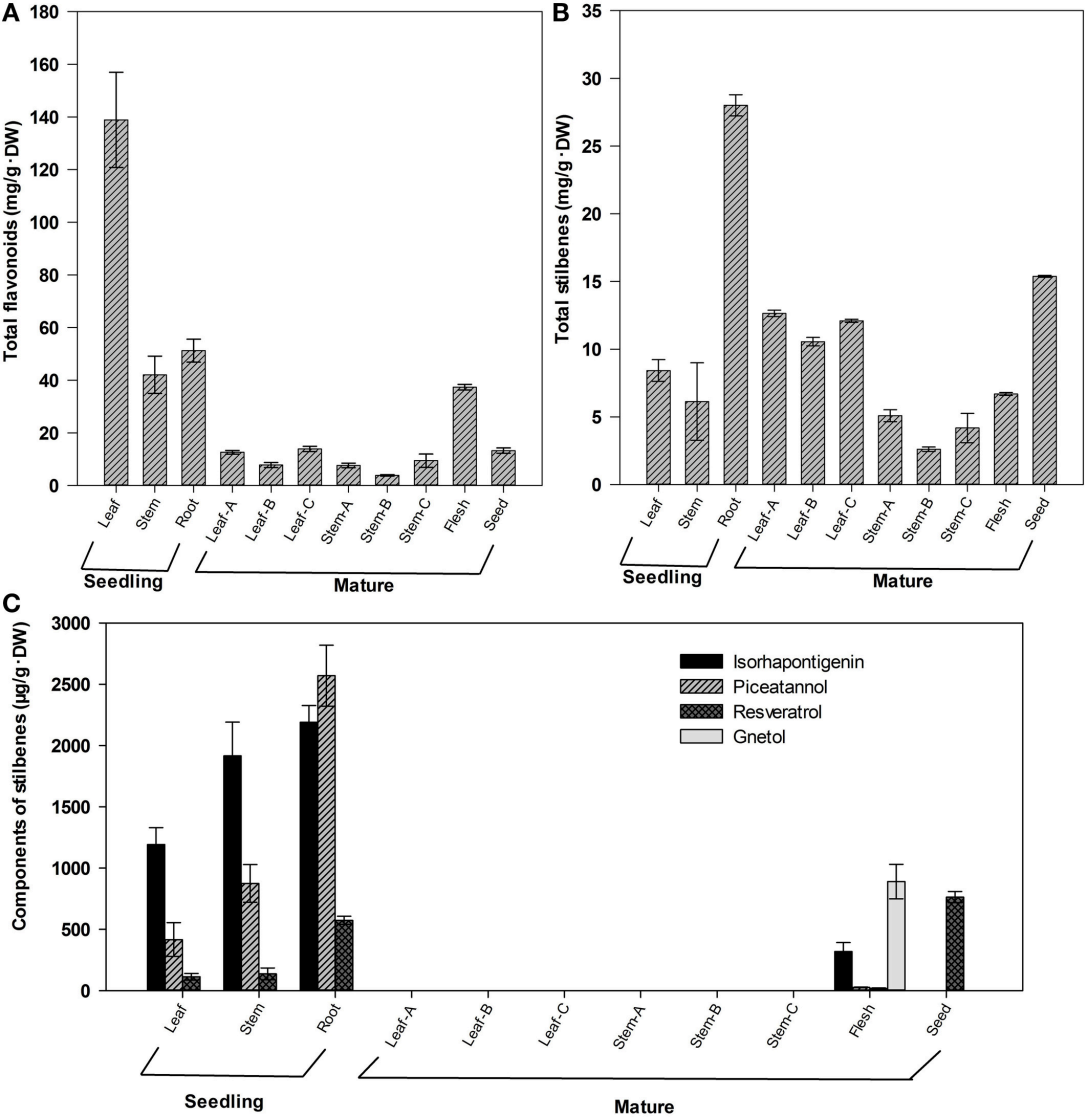
**Contents of total flavonoids and total stilbenes in different tissues of *Gnetum parvifolium***. **(A)** Total flavonoids; **(B)** Total stilbenes; **(C)** Component of stilbenes. Vertical bars represent the mean ±SD of four separate experiments. In figure, Leaf, Stem, and Root were collected from 1-year old seedlings; Leaf/Stem-A, -B, and –C were three stages of leaves/stems from young to old, respectively, collected from mature trees in September; Flesh and Seed were fruit flesh (aril) and seeds, respectively.

Our previous studies showed that the seeds are rich in total flavonoids and stilbenes in *G. parvifolium* (Lan et al., 2014). In this study, the tissues from young seedlings and mature trees were further used to investigate the distribution of flavonoids and stilbenes in *G. parvifolium*. The flavonoids were present in all tissues of young seedlings, fruit flesh (aril), and seeds (Figure 1A). Their content was highest in leaves (138.9 mg/g·DW), followed by roots and stems of seedlings, and fruit flesh, with the contents between 37.4–51.2 mg/g·DW. Other tissues had relatively low levels of flavonoids: less than 13.8 mg/g·DW in leaves and stems of mature trees, and seeds. However, stilbenes were highly accumulated in roots of young seedlings, leaves of mature trees and seeds (Figure 1B). The content of stilbenes in roots of seedling was 28.0 mg/g·DW, followed by seeds (15.4 mg/g·DW), and leaves of mature trees (10.6–12.6 mg/g·DW). Moreover, four specific stilbene components (resveratrol, piceatannol, isorhapontigenin, and gnetol) were identified in the selected tissues (Figure 1C), and the former three components were found in young seedlings. Roots of seedlings were most rich in resveratrol, isorhapontigenin, and piceatannol with 573.7, 2189.3, and 2569.2 μg/g·DW, respectively. Additionally, resveratrol (763.1 μg/g·DW) was also found in seeds, while gnetol was only found in fruit flesh (890.4 μg/g·DW). Surprisingly, these four stilbenes were not detected in leaves and stems of mature trees. These results indicated that flavonoids and stilbenes can accumulate in different tissues of *G. parvifolium* at relatively high concentrations, although we could not detect any specific components of flavonoids in the present study. Therefore, to decipher the biosynthetic pathways of these metabolites, especially stilbenes, we performed transcriptome sequencing from a pooled RNA samples from various tissues as described in the present study.

### Transcriptome sequencing and assembly

To globally and comprehensively cover the transcriptome of *G. parvifolium*, a cDNA library was prepared from pooled samples and sequenced. After a stringent quality check, 77,072,678 raw reads (9.4 Gb), with an average GC content of 45.0% (Supplementary Table S1A), were generated from Illumina HiSeq/MiSeq. The number of clean reads was 74,947,178 (97.2%; Supplementary Figure S1A). After trimming and assembling, 196,728 transcripts and 94,816 unigenes were generated (Supplementary Figure S1B; Supplementary Table S1B). Their length distributions were shown in Supplementary Figures S1C,D. The N50 value of the unigenes was 1,397 bp, and their average length was 724 bp (ranging from 201 to 17,599 bp; Supplementary Table S1B); 59.3% of all unigenes were longer than 300 bp (Supplementary Figure S1B). The relative expression level of each unigene was estimated by using the FPKM approach. The expression of unigenes ranged from 0 to 16,045.57 FPKM with an average of 8.24 FPKM. Of the 94,816 unigenes, 82,926 (87.5%) had a very low expression level of less than 10 FPKM (Supplementary Table S2).

### Functional annotation and categorization

**Figure 2:**
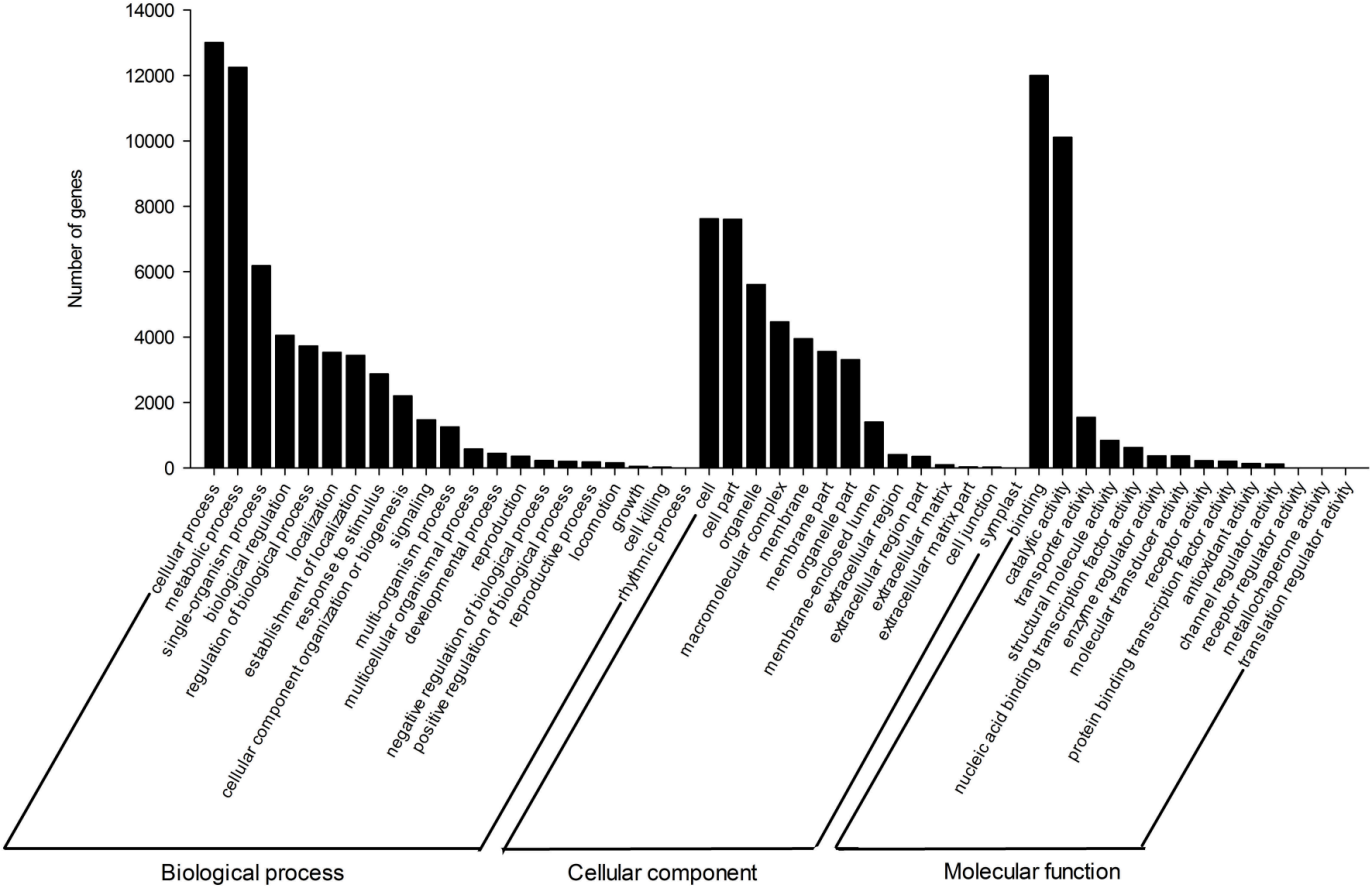
**Functional annotation of unigenes based on gene ontology (GO) categorization**. Main functional categories in the biological process, cellular component, and molecular functions relevant to plant physiology. Bars represent the numbers of *Gnetum parvifolium* assignment proteins with BLASTX matches to each GO term. One unigene may be matched to multiple GO terms.

For the verification and annotation of the assembled unigenes, all the assembled sequences were initially searched against the NR and Swiss-Prot protein databases, using the BLASTX program. Among the 94,816 unigenes, 21,308 (22.5%) had significant hits in the NR database, and 15,359 (16.2%) had significant matches to proteins in the Swiss-Prot database (Supplementary Tables S1C, S3). In this study, 21,498 (22.7%) unigenes were assigned to one or more GO terms (Supplementary Table S1C), which were then classified into three main categories, (i) biological process, (ii) cellular component, and (iii) molecular function clusters, and they were further distributed across 49 sub-categories (Figure 2 and Supplementary Table S4). Among biological processes, candidate genes involved in metabolic and cellular processes were highly represented.

**Figure 3:**
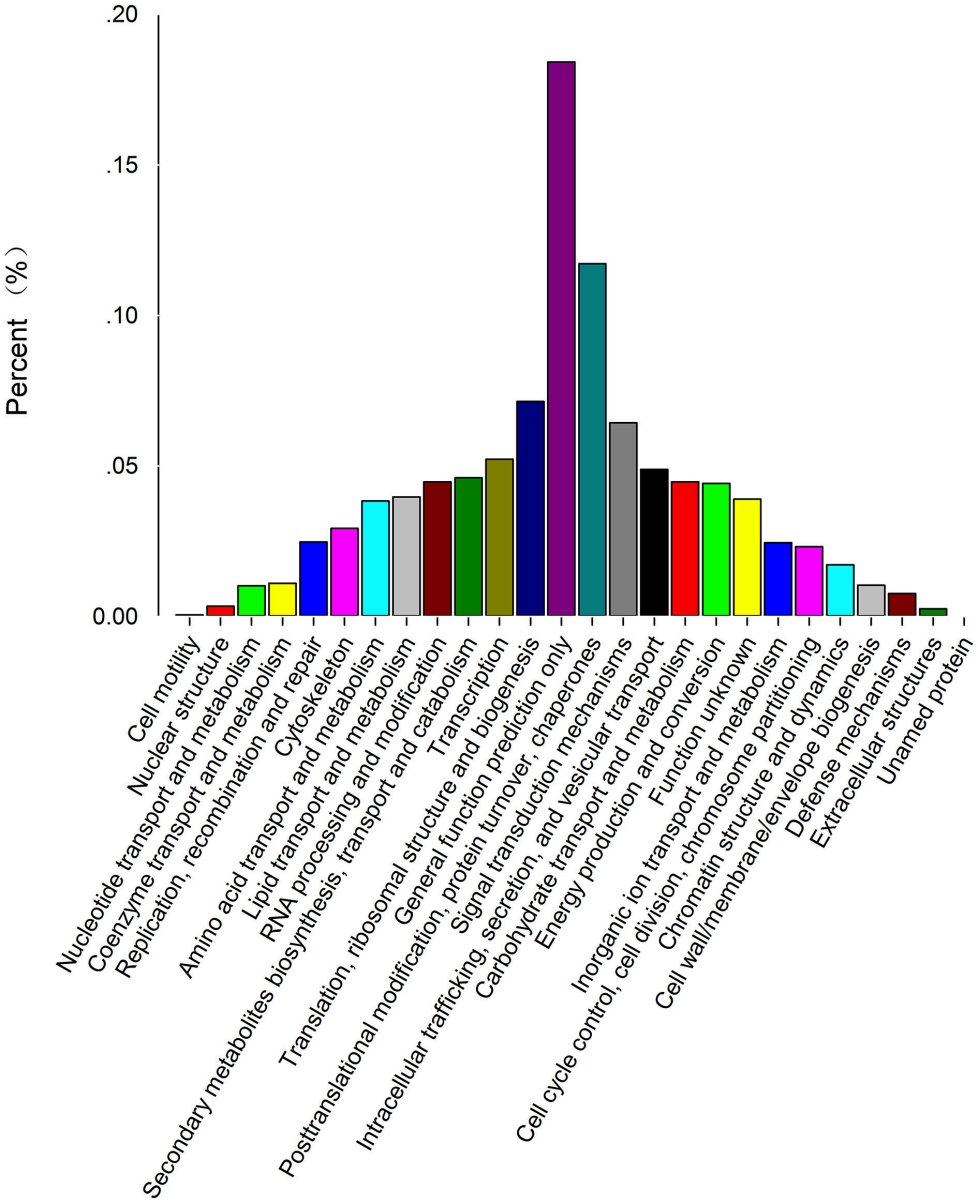
**KOG classification of unigenes**. Eight thousand six hundred forty-three unigenes with Nr hits were grouped into 26 KOG terms. The Y-axis represents the percentage of all unigenes.

In addition, all unigenes were subjected to a search against the KOG database. Eight thousand six hundred forty-three were assigned to KOG classifications (Figure 3; Supplementary Table S1C). Among the 26 KOG categories, the “general function prediction” represented the largest group (1787, 20.7%), followed by “posttranslational modification protein turnover, chaperones” (1137, 13.2%), “translation, ribosomal structure, and biogenesis” (693, 8%), “signal transduction mechanisms” (624, 7.2%) and “transcription” (507, 5.9%). Among 521 candidate unigenes (6.0%) found in the categories “secondary metabolites biosynthesis, transport, and catabolism” and “defense mechanisms,” most (450, 5.2%) were involved in secondary metabolite biosynthesis (Supplementary Table S5).

### Analysis of metabolic pathways by kyoto encyclopedia of genes and genomes (KEGG)

**Figure 4:**
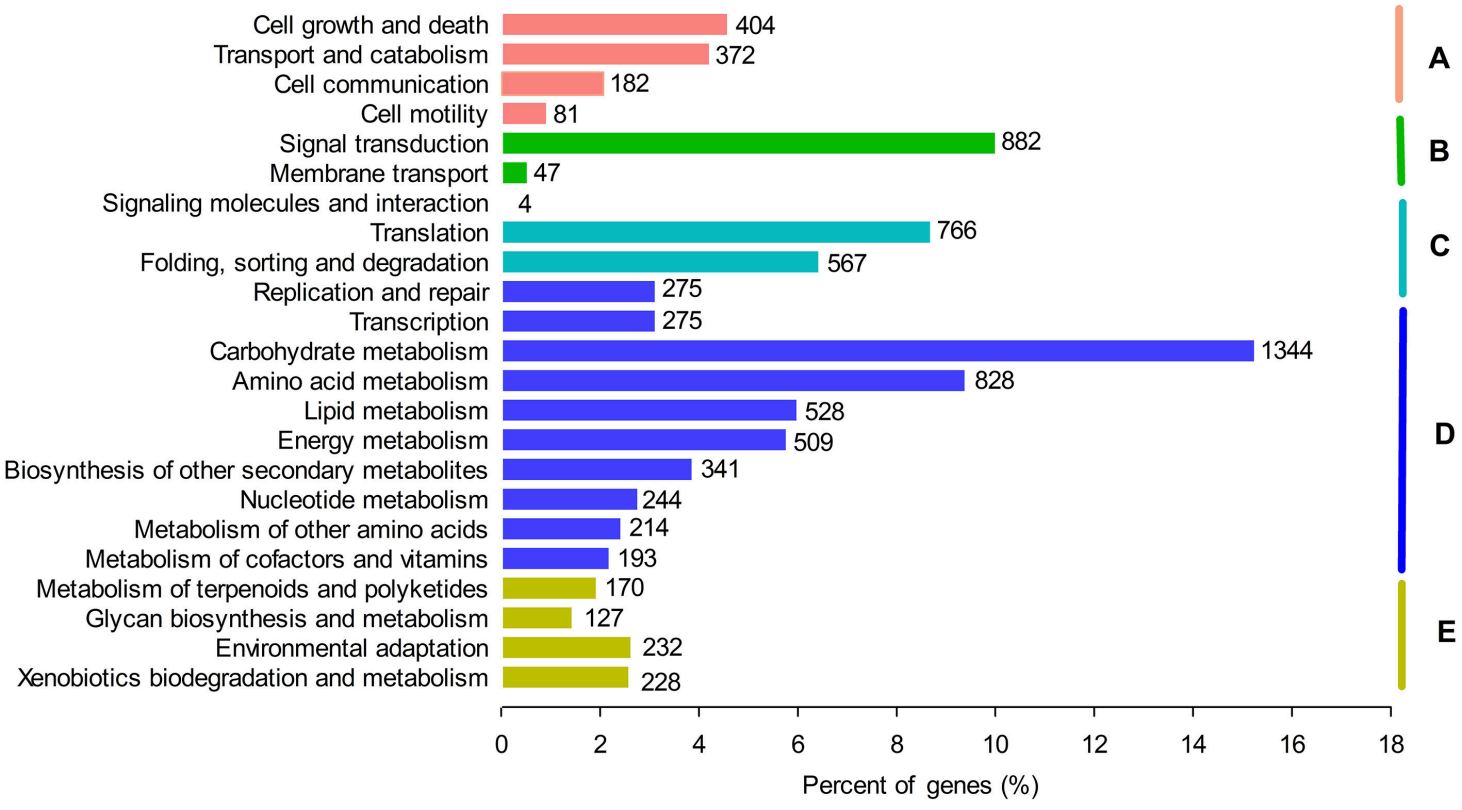
**Functional annotation of unigenes based on KEGG categorization**. Main functional categories are the Cellular Processes **(A)**; Environmental Information Processing **(B)**; Genetic Information Processing **(C)**; Metabolism **(D)**; and Organismal Systems **(E)**. Bars represent the numbers of *Gnetum parvifolium* assignments of unigenes with BLASTX matches to each KEGG term.

To further investigate the medicinal or healthy values of *G. parvifolium*, we analyzed all unigenes using the KEGG database. We identified 131 pathways involved in metabolism of plants from this species, representing plant biochemical pathways, metabolic processes, and some important secondary metabolite biosynthesis pathways (Figure 4; Supplementary Table S6A). Most of the metabolism pathways (35.9%) were related to certain important secondary metabolites, including phenylpropanoids, flavonoids (flavone, flavonol, and flavonoid), stilbenoids (stilbenoid, diarylheptanoid, and gingerol), and also alkaloids, terpenoids, and polyketides (Supplementary Table S6B). The candidate unigenes involved in the biosynthetic pathways of phenylpropanoids, flavonoids, and stilbenoids, most of which had more than 50% identities with functionally validated enzymes in top blast rank (Supplementary Table S7), were further investigated in more detail.

### Identification of candidate genes involved in phenylpropanoid pathway

In this study, we identified 126 candidate unigenes across 14 gene families associated with phenylpropanoid pathway from *G. parvifolium* transcriptome by KEGG analysis (Table 1; Supplementary Figure S2). In the initial three steps, we obtained 15 key candidate unigenes including seven *PAL*s, two *C4H*s (*CYP73A*s), and six *4CL*s. The enzymes encoded by these genes catalyze a series of reactions to form cinnamoyl-CoA or p-coumaroyl CoA, directly as the substrate of the biosynthetic pathways of flavonoids and stilbenoids. We also found six *HCT*s (shikimate O-hydroxycinnamoyltransferase) and five *C*3′*H*s (p-coumarate 3-hydroxylase). Enzymes encoded by these genes can synthesize caffeoyl CoA, which can be further catalyzed by six *CCOAMT*s (caffeoyl CoA 3-O-methyltransferase) to synthesize feruloyl CoA or sinapoyl CoA. These precursors undergo different catalyzing pathways to form various kinds of lignins. In these enzyme reaction processes, we identified several candidate key genes, including one *CCR* (cinnamoyl-CoA reductase), 14 *CAD*s (cinnamyl alcohol dehydrogenase), and 72 *PRX*s/*PRD*s/*KatG*s (peroxidase/peroxiredoxin/catalase-peroxidase). Additionally, we also found two candidate *UGT72E*s (coniferyl-alcohol glucosyltransferase), which can catalyze the different substrates to form their glycosides, such as coniferin and syringin. Thus, our analysis provided detailed information on this pathway in *G. parvifolium*, particularly concerning candidate genes involved in the biosynthesis of precursors for flavonoids and stilbenoids.

**Table 1:** **Candidate unigenes involved in biosynthetic pathways of phenylpropanoids and flavonoids in the transcriptome of *Gnetum parvifolium***.

**KEGG**	**Gene**	**Gene name**	**Number**	**Identities (%)**
Phenylpropanoids	*PAL*	Phenylalanine ammonia-lyase	7	61.3–82.6
	*C4H*	Cinnamic acid 4-hydroxylase	2	67.5–82.0
	*4CL*	4-coumarate coenzyme A ligase	6	50.3–69.0
	*CCR*	Cinnamoyl-CoA reductase	1	78.1
	*CAD*	Cinnamyl alcohol dehydrogenase	14	52.4–85.7
	*KatG*	Catalase-peroxidase 1	2	77.9–76.5
	*UGT72E*	Coniferyl-alcohol glucosyltransferase	2	45.6–54.0
	*HCT*	Shikimate O-hydroxycinnamoyltransferase	6	43.1–62.8
	*C*3′*H*	p-Coumarate 3-hydroxylase	5	48.6–71.7
	*CCOAMT*	Caffeoyl-CoA O-methyltransferase	6	52.4–87.6
	*F5H*	Ferulate 5-hydroxylase	3	47.4–52.2
	*REF1*	Aldehyde dehydrogenase	2	74.2–89.0
	*PRD*	1-Cys peroxiredoxin	2	56.0–64.7
	*PRX*	Peroxidase	68	44.2–79.6
Flavonoids	*CHS*	Chalcone synthase	18	55.8–79.1
	*CHI*	Chalcone isomerase	1	42.9
	*F3H*	Flavanone 3-hydroxylase	2	41.6–43.0
	*FLS*	Flavonol synthase	2	43.0–46.2
	*DFR*	Dihydroflavonol-4-reductase	7	41.5–67.4
	*F*3′*H*	Flavonoid 3′-hydroxylase	8	41.4–57.4
	*F*3′5′*H*	Flavonoid 3′, 5′-hydroxylase	1	55.3
	*LAR*	Leucoanthocyanidin reductase	1	56.1
	*ANR*	Anthocyanidin reductase	2	47.7–55.2
	*CCOAMT*	Caffeoyl-CoA O-methyltransferase	6	52.4–87.6
	*F3OMT*	Flavonol 3-O-methyltransferase	6	40.7–59.0

*Unigenes with more than 40% identities with functionally validated enzymes were selected from Supplementary Table S7*.

### Identification of candidate genes involved in flavonoid pathway

In the present study, we identified 54 candidate unigenes across 11 gene families associated with the flavonoid pathway in *G. parvifolium* transcriptome (Table 1; Supplementary Figure S3), which were involved in three main sub-pathways derived from the different intermediates, cinnamoyl-CoA and *p*-coumaroyl CoA (Supplementary Figure S3). In the upstream pathway, we found 18 *CHS*s and one *CHI* (chalcone isomerase) involved in the two-step condensation to produce the basic skeletons including naringenin, pinocembrin, and liquiritigenin. Following the core sub-pathway of naringenin, we identified two *F3H*s (flavanone 3-hydroxylase), eight *F*3′*H*s (flavonoid 3′-hydroxylase), and one *F*3′5′*H* (flavonoid 3′, 5′-hydroxylase), yielding eriodictyol and dihydrokaempferol, repectively. The latter two genes encoded enzymes which also can contribute to the production of dihydroquercetin, dihydrotricetin, and other flavonoids. In the downstream pathway, we identified seven *DFR*s (flavanone 4-reductase), two *FLS*s (flavonol synthase), one *LAR* and two *ANR*s, producing numerous different kinds of flavonoids as shown in Supplementary Figure S3. Our findings thus indicate that the identified candidate genes involved in flavonoid pathway in *G. parvifolium* could resemble a complex metabolic grid rather than a linear pathway.

### Identification of candidate genes involved in stilbenoid pathway

**Figure 5:**
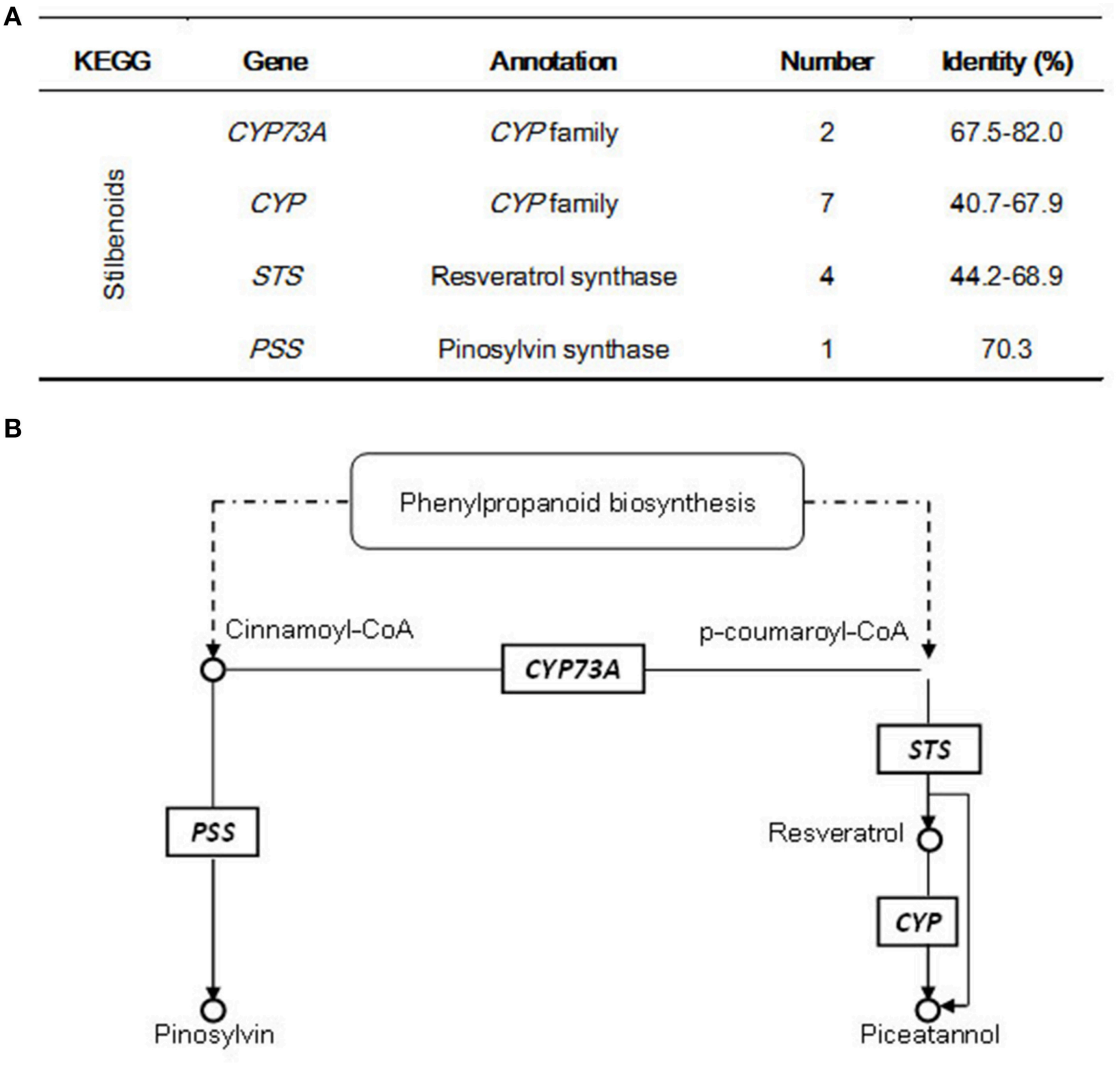
**Candidate unigenes involved in stilbenoid biosynthesis in *Gnetum parvifolium*. (A)** Annotatin of candidate genes; **(B)** Biosynthetic pathway with identified candidate genes; referred from Hammerbacher et al. (2011), and KEGG pathway “Stilbenoid, diarylheptanoid, and gingerol biosynthesis” (http://www.kegg.jp/kegg-bin/show_pathway?ko00945+K00517).

Consistent with the determination of stilbenoid components in different plant tissues (Figures 1B,C), 14 candidate unigenes were found to be involved in stilbenoid pathway, which has two main sub-pathways in *G. parvifolium* (Figure 5). We identified five stilbene synthase (STS)-related genes including four *STS*s (normally named resveratrol synthase) and one pinosylvin synthase (PSS; stilbene synthase isoform) gene, which encode key and rate-limiting enzymes in the biosynthesis of stilbenoids. We found that *STS* shares the same substrate, p-coumaroyl-CoA, with chalcone biosynthesis (as shown in Supplementary Figure S2) to synthesize resveratrol. Resveratrol, as a direct precursor, could be further catalyzed by hydroxylation into piceatannol (a resveratrol analog) by some members of *CYP* gene family (cytochrome P450 genes; seven candidate unigenes are listed in Supplementary Table S12); and the latter stilbene can also be directly formed by the catalyzation of *STS* encoded enzyme (Figure 5B). In the other sub-pathway, cinnamoyl CoA, which does not need to be converted to p-coumaroyl-CoA by *C4H*s (*CYP73A*s) (two candidate unigenes), can be used directly as a substrate to synthesize pinosylvin by the enzyme encoded by another *STS*-related gene, *PSS* (Figure 5B).

### Expression patterns of candidate genes involved in stilbenoid pathway in different tissues

**Figure 6:**
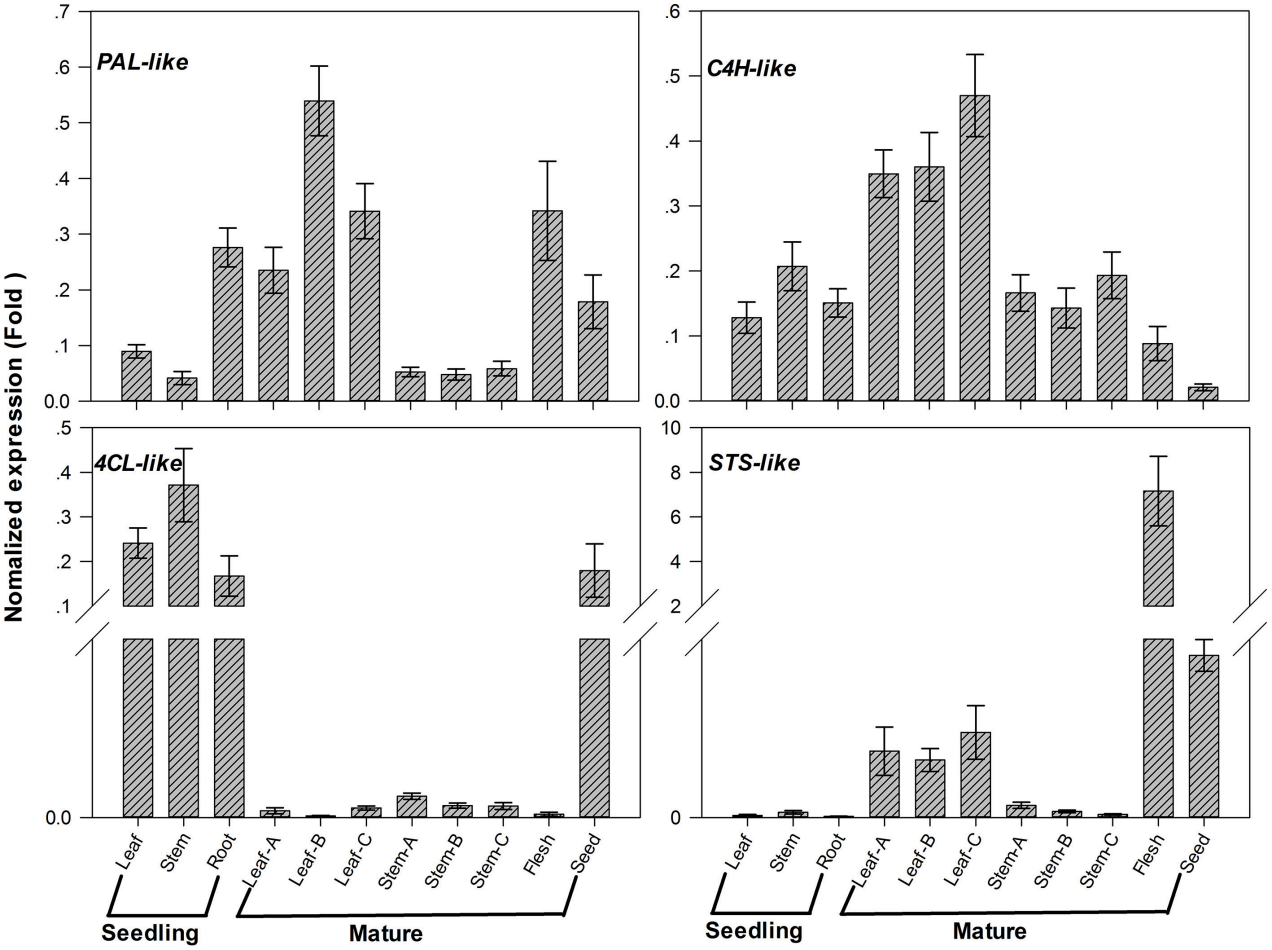
**Expression patterns of candidate genes involved in stilbenoids biosynthesis in different tissues from *Gnetum parvifolium***. Vertical bars represent the mean ±SD of four separate experiments. In figure, Leaf, Stem, and Root were collected from 1-year old seedlings; Leaf/Stem-A, -B, and -C were three stages of leaves/stem from young to old, respectively, collected from mature trees in September; Flesh and Seed were fruit flesh (aril) and seeds, respectively. *PAL-like*: comp81110_c0; *C4H-like*: comp90938_c0; *4CL-like*: comp94230_c0; *STS-like*: comp550004_c0.

Analysis of secondary metabolites showed that the total stilbenes were distributed in different tissues of *G. parvifolium* (Figures 1B,C). We focused on analyzing expression patterns of four candidate unigenes (*PAL-, C4H-, 4CL-*, and *STS-like* genes) associated with stilbenoids biosynthesis by using specific primers (Supplementary Table S8). The upstream candidates *PAL-, C4H-*, and *4CL-like* showed different expression patterns (Figure 6). *PAL-like* expression was higher in roots of seedlings, leaves of mature trees, and seeds besides fruit flesh, while another *PAL-like* (comp69381_c0) showed highest expression in fruit flesh (Supplementary Figure S4A); *C4H-like* expression was higher in leaves of mature trees than that in other parts; *4CL-like* showed drastically higher expression in young seedlings and seeds, than in other tissues. The candidate *STS-like*, as probably a key and limiting gene encoding enzyme to produce resveratrol, showed expression pattern similar to *PAL-like* (comp69381_c0) (Supplementary Figure S4A). *STS-like* had especially low expression level in young seedlings; whereas it showed considerably higher expression in the fruit flesh than those in any other tissues; additionally, its expression was also high in seeds and leaves of mature trees.

### Candidate genes involved in stilbenoid pathway induced by high temperature and UV-C

**Figure 7:**
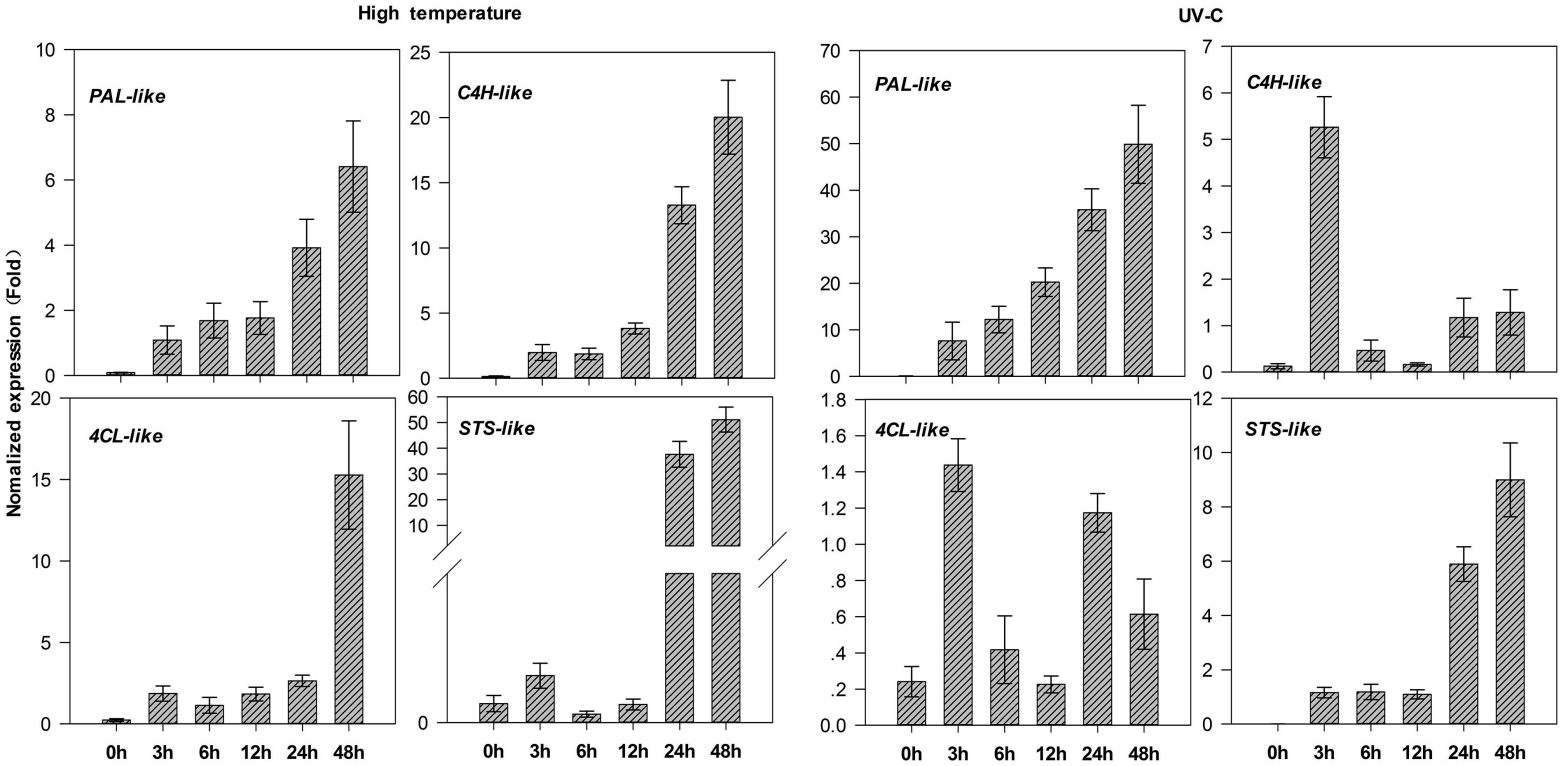
**Expression patterns of candidate genes involved in stilbenoid biosynthesis in leaves of *Gnetum parvifolium* seedlings under stresses**. Young seedlings were treated with high temperature (40°C)/UV-C, respectively, for 0, 3, 6, 12, 24, and 48 h. Vertical bars represent the mean ±SD of four separate experiments. *PAL-like*: comp81110_c0; *C4H-like*: comp90938_c0; *4CL-like*: comp94230_c0; *STS-like*: comp550004_c0.

Application of high temperature and UV-C obviously strongly induced the expression of four candidate genes (*PAL, C4H-, 4CL-*, and *STS-like* genes), and another *PAL-like* (comp69381_c0) (Supplementary Figures S4B,C); moreover, their expression levels increased drastically with the extension of stress time, although *C4H-* and *4CL-like* genes showed some fluctuations under UV-C treatment (Figure 7). Interestingly, *STS-like* gene showed especially low or almost no expression before 12-h treatments, while its expression level was enhanced more than 119.4 and 996.7 folds after 24-h treatments under high temperature and UV-C, respectively, compared to controls (0 h). These results thus indicate that these candidate genes associated with stilbene biosynthesis had significant responses to stress conditions.

**Figure 8:**
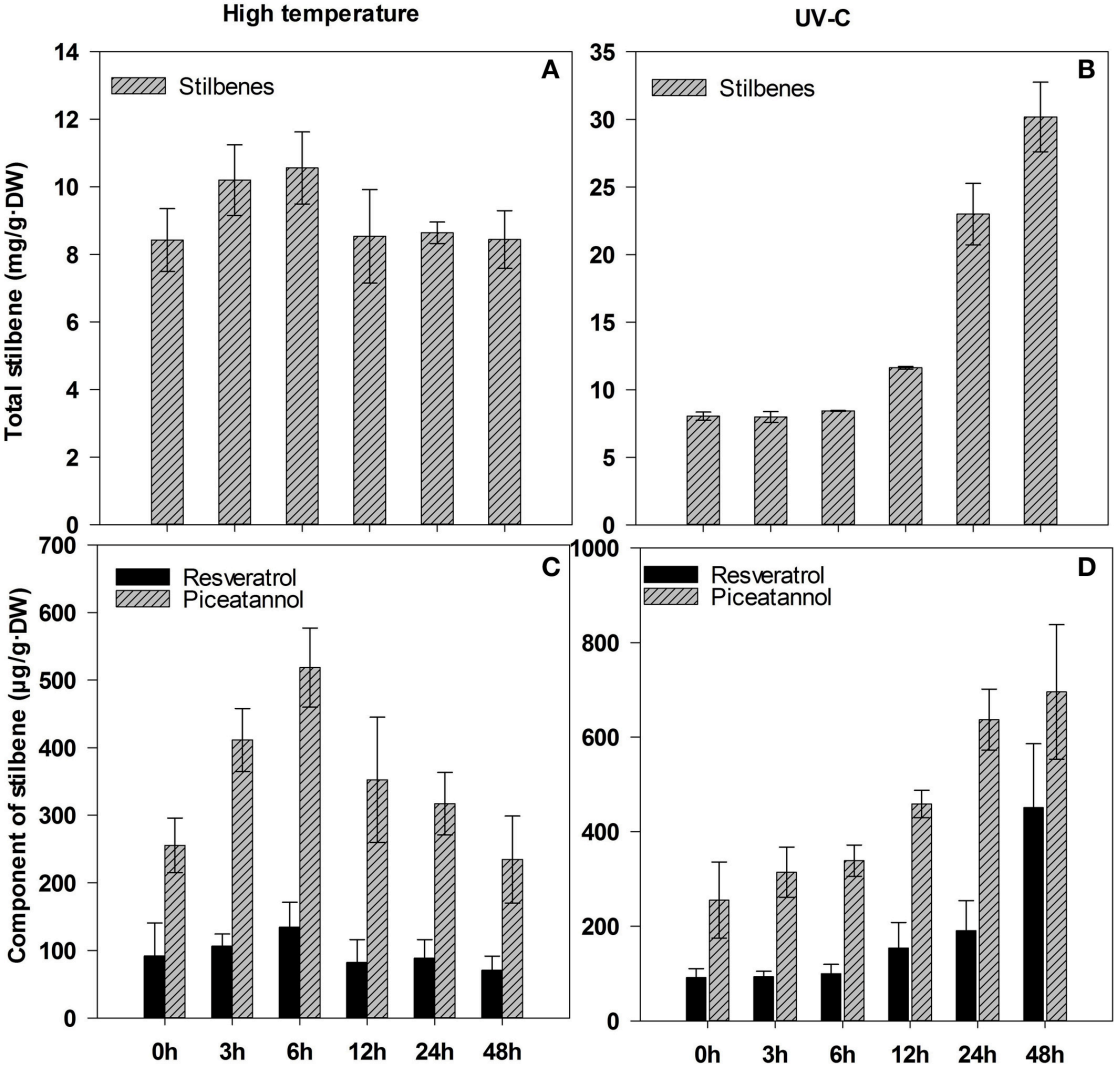
**Content of total stilbenes and its components in leaves of *Gnetum parvifolium* seedlings under stresses**. **(A)** and **(B)** report total stilbenes under high temperature and UV-C, respectively; **(C)** and **(D)** report component of stilbenes under high temperature and UV-C, respectively. Young seedlings were treated with high temperature (40°C)/UV-C, respectively, for 0, 3, 6, 12, 24, and 48 h. Vertical bars represent the mean ±SD of four separate experiments.

Correspondingly, total contents of stilbenes increased in tissues under UV-C stress, we detected 2.9-fold increase of the contents at 24 h compared to controls (0 h); high temperature, however, had no significant effect on the accumulation of total stilbenes between 8.4–10.5 mg/g·DW (Figures 8A,B). Further quantification by HPLC showed that high temperature had no obvious influence on biosynthesis of resveratrol compared to controls (0 h), but induced a considerable increase in piceatannol, which reached the highest concentration of 518.4 μg/g·DW at 6 h, a 2.0-fold increase compared to control (0 h) (Figure 8C). However, UV-C stimulated obvious increase in both stilbenes with the extension of treatment time. Especially after 24-h UV-C stimuli, the accumulation of resveratrol was between 190.8 and 450.4 μg/g·DW, and that of piceatannol was between 636.8 and 695.6 μg/g·DW, over 2.1- and 2.5-fold increase, respectively, compared to control (0 h) (Figure 8D). These results showed the accumulation of resveratrol and piceatannol was consistent with the *STS-like* expression under UV-C stress, while there was no obvious relation with the expression of *STS-like* gene under high temperature.

### SSRs involved in secondary metabolism from *G. parvifolium* transcriptome

In this study, we identified SSRs from 71 unigenes associated with secondary metabolism (Supplementary Table S9), based on SSRs identified in the whole *G. parvifolium* transcriptome. The identified SSRs included 70.6% tri-nucleotides, followed by dinucleotide (23.8%), and tetra-, penta-, and hexa-nucleotide repeats with low percentages (Supplementary Table S10; Supplementary Figure S5). Of these, 13 SSRs motifs were linked with unique sequences encoding enzymes involved in flavonoid and stilbenoid biosynthetic pathways (Table 2), including two tetra-nucleotide repeats, six tri-nucleotide repeats, and five dinucleotide repeats. The unique sequence-derived markers generated in this study represent a valuable genetic resource for future investigation of secondary metabolism in *Gnetum*.

**Table 2:** **SSR motifs in putative genes involved in biosynthetic pathways of flavonoids and stilbenoids**.

**Gene**	**Unigene**	**Motif**	**Size (bp)**	**End (bp)**	**Start (bp)**
*CHS*	comp95326_c1	(CGTC)6	24	1181	1204
*C4H*	comp90938_c0	(TA)10	20	1	20
*F*3′*H*	comp63646_c0	(ATT)5	15	73	87
*F*3′5′*H*	comp90819_c0	(CTG)5	15	966	980
*DFR*	comp94260_c1	(AAT)6	18	520	537
*F3OMT*	comp87212_c0	(AG)7	14	1732	1745
	comp89704_c0	(ACA)5	15	572	586
	comp94680_c0	(AAT)5	15	1761	1775
*CYP73A*	comp90938_c0	(TA)10	20	1	20
*CYP*	comp92132_c0	(AG)6	12	1874	1885
	comp85497_c0	(GT)6	12	3072	3083
	comp96993_c0	(CAG)5	15	1695	1709
	comp90467_c0	(CGTG)6	24	2732	2755

*Repeats of mononucleotides were excluded from this table*.

## Discussion

High-throughput mRNA sequencing technology is a fast, efficient and cost-effective way to characterize the transcriptome, and provides ready access to high resolution transcriptome information to an extent that was once unimaginable (Martin et al., 2013). Up to now, only 10,728 EST sequences can be found by searching NCBI databases (the search was performed on 22.01.2016) in the important medicinal plant, *Gnetum*, which has been reported to be rich in anticancer, antioxidant, and antibacterial components, such as flavonoids and stilbenoids (Fang et al., 2013). The present study aimed to characterize the metabolic pathways of some important bioactive compounds via a comprehensive in-depth investigation of the *G. parvifolium* transcriptome using RNA-seq. To generate data for an overview of the plant genetic composition, we used tissue samples for RNA preparation from different organs of this species, which were selected to acquire as a comprehensive coverage of organs as possible. We obtained 27,722 unigenes in *G. parvifolium* after *de novo* assembly, which were annotated in at least one database (Supplementary Table S1C). The number was roughly similar to the one from transcriptome of *Picea balfouriana*, where 22,295 unigenes (Li et al., 2014) represented 78.6% of the 28,354 genes in the *P. abies* genome (Nystedt et al., 2013). Meanwhile, 55,088 unigenes were annotated in the transcriptome of angiosperm *Camellia sinensis* (Shi C. Y. et al., 2011). The reason for this difference might be that *Gnetum* is more closely related to conifers than to flowering plants (Winter et al., 1999). Genomes of angiosperms are expected to comprise larger numbers of unigenes because of multiple whole genome duplications. On the other hand, few conifers have been subjected to whole genome sequencing so far (except for *P. abies*; Nystedt et al., 2013). Therefore, in our study the large number of currently non-annotated unigenes might represent gymnosperm-specific (Hou et al., 2011) or *Gnetum*-specific genes, although sequencing errors remained unavoidable under stringent quality control (Supplementary Table S1A; Quail et al., 2012).

In *G. parvifolium*, the functions of 27,722 annotated unigenes were inferred by COG, GO, and metabolic pathways analyses. The identification of these candidate unigenes involved in the biosynthesis of important secondary metabolic compounds represents an opportunity to learn more about the global regulation networks of secondary metabolism at the transcriptome level in Gnetophyta. The analysis of KOG classifications and KEGG pathways led to the identification of genes related to secondary metabolites, particularly some important bioactive compounds (Table 1; Figure 5; Supplementary Figures S2, S3). As one of the main goals of this study, many candidate genes involved in the biosynthetic pathways of flavonoids (Table 1; Supplementary Figure S3) and stilbenoids (Figure 5), which are the important derivatives of phenylalanine metabolism, were identified in *G. parvifolium* by KEGG pathways analysis. Furthermore, most of candidate genes involved in the pathways of phenylpropanoids, flavonoids, and stilbenoids showed high homologies to their functionally validated enzymes (Supplementary Table S7), which indicated the functions of the analyzed candidates were identified reliably.

Flavonoids are widely distributed secondary metabolites with different metabolic functions in plants (Falcone et al., 2012). Flavonoids are not only vital for plant growth, development and protection, but also are beneficial to human health, via their anti-inflammatory, antioxidant, antimicrobial, and anticancer properties (Mouradov and Spangenberg, 2014). We identified 11 families of genes (54 candidate unigenes) in the biosynthetic pathway of flavonoids in *G. parvifolium* (Table 1; Supplementary Figure S3; Supplementary Table S6). These candidates represented most genes in this network, although only a few candidates were identified in the pathway of flavone and flavonol biosynthesis. Our findings were consistent with previous determinations of these compounds, including their components (for the details, see the Introduction). Previous studies from our group have also shown that this species has high contents of total flavones, resveratrol, isorhapontigenin, and gnetol in seeds (Lan et al., 2013, 2014). In our study, several specific representatives of stilbenes were identified in a variety of plant tissues, although no specific flavonoid components could be detected (Figure 1). In our further work, we would like to verify the expression of genes associated with flavonoid pathway and identify specific flavonoids and their amounts in the different tissues under normal and stress conditions to explain why *Gnetum* is so rich in flavonoids.

Different variants of stilbenes are abundant in plants from *Gnetum*, similar to other stilbene-producing plants. Therefore, physiological and molecular research on *Gnetum* could lead to important discoveries of new bioactive and health-related compounds. In this study, we found five candidate unigenes that represented *STS* related genes (encoding stilbene synthases and pinosylvin synthase) and 51 candidate unigenes matched with the term “chalcone and stilbene synthases” in at least one of the used databases (Supplementary Table S11). These potential genes might be related to stilbene biosynthesis, but this assumption requires further verification. As shown in Supplementary Figure S2, both CHS and STS use p-coumaroyl-CoA and malonyl-CoA as substrates, and they synthesize the same linear tetraketide intermediate. The difference is that STS uses a specific cyclization mechanism involving decarboxylation to form the stilbene backbone. STS proteins share extensive amino acid sequence identity with CHS (Parage et al., 2012), and phylogenetic analysis with *STS* and *CHS* gene families has shown that *STS*s may have evolved from *CHS*s (Tropf et al., 1994). In most stilbene-producing plants, *STS* genes form small families of closely related paralogs (Parage et al., 2012). For example, the genome of *Pinus sylvestris* contains a small family of four *STS* genes (Preisig-Muller et al., 1999); three *STS* genes have been characterized in Japanese *P. densiflora* (Kodan et al., 2002); and one *STS* gene was identified in sorghum genome (Yu et al., 2005). By contrast, the grapevine genome has a large multigene family, with an estimated number of *STS* genes ranging from 21 to 43 (Jaillon et al., 2007; Velasco et al., 2007). For *Gnetum*, a hundred different kinds of stilbenoids have been reported (Wang and Liang, 2006; Shi S. Q. et al., 2011; Riviere et al., 2012). However, in this study we found only five candidate synthase-related genes (four *STS*s and one *PSS*) in *Gnetum*. One explanation might be that the used samples were collected under normal conditions, while stilbenes are a type of phytoalexins, generally responding to stressful environmental cues, such as high temperature, restricted nutrition, microbial elicitors, and UV light (Di et al., 2012). This explanation was supported by our quantification of stilbenes in *G. parvifolium* exposed to high temperature and UV-C (Figure 8).

Gene expression levels determined by qRT-PCR showed that four selected candidate genes involved in stilbene biosynthesis were all highly expressed in leaves of mature tree, and fruit flesh and seeds, especially for *STS-like* gene (Figure 6). However, *STS-like* was very weakly expressed in young seedlings, whereas its expression was stimulated drastically under high temperature and UV-C (Figure 7). This finding could benefit the understanding of *Gnetum* adaptation to the climate of tropical and subtropical areas with high temperature and strong UV radiation. Additionally, it is well known that the *STS* gene encodes a key and rate-limiting enzyme to produce backbone stilbene, resveratrol (Watts et al., 2006; Katsuyama et al., 2007), which potentiates the anti-tumor effects of different cancer therapies (Gwak et al., 2016). Resveratrol and its derivatives from *Gnetum* are involved in suppression of the multiple angiogenesis-related endothelial cell functions and/or tumor angiogenesis (Kunimasa et al., 2011). Interestingly, in Central Africa and Southeast Asia, the young leaves and fruits from some *Gnetum* species, such as *G. africanum* and *G. gnemon*, are consumed widely as healthy vegetables and nuts (Isong et al., 1999). Combined with identification of SSR markers associated with secondary metabolism (Table 2; Supplementary Table S9), which are highly informative and widely used in evolution and breeding studies (Liu et al., 2012), our results strongly suggest that young *Gnetum* seedlings might be cultivated under optimal stress conditions in order to get stilbene-rich vegetables. More work is however necessary to understand the so far lacking in our study link between expression patterns of *STS-like* gene and accumulation of resveratrol in different plant tissues under stressful conditions of high temperature.

In conclusion, the lack of a reference genome for *Gnetum* has made it difficult to estimate the number of genes and predict their potential functions in this phylogenetically distinct group of plants. Here, a large number of candidate unigenes could be matched with unique known proteins in public databases, indicating that the sequencing project identified a substantial proportion of gene resources of *G. parvifolium*. These candidate genes may perform specific roles in *Gnetum* and may be quite divergent from those of other plant species. Therefore, our study can (i) improve considerably understanding of secondary metabolism in this evolutionary diverged lineage of seed plants, and (ii) provide reference sequences for evolutionary analyses of metabolomes in both angiosperms and gymnosperms. Moreover, the studies on pathways of flavonoids and stilbenoids would benefit understanding of environmental adaptation and economic utilization in *Gnetum*. Thus, the transcriptome sequence generated in this study represents a valuable resource for further research, such as functional genomics, evolutionary analyses, and breeding of plants that are rich in bioactive components.

## Deposited data

The RNA-seq datasets generated by using Illumina-Solexa platform are available from the NCBI Sequence Read Archive database (SRA; http://www.ncbi.nlm.nih.gov/sra) under experiment number accession SRX1133345. The cDNA libraries were obtained from different tissues including seeds (five stages from inflorescence to mature seed, including fruit); germinated seeds (four stages based on the size of the embryo); young inflorescences; and leaves, roots, stems, shoot apices from mature trees and young seedlings.

## Author contributions

Manuscript draft: ND, SS; analyzing data: ND, EC, SS; experiment: ND, EC, ML, JJ, JL, JM, LC; ND, EC, SS, and IB contributed to writing the text; conception and supervision of the research: ZJ and SS.

### Conflict of interest statement

The authors declare that the research was conducted in the absence of any commercial or financial relationships that could be construed as a potential conflict of interest. The reviewer GM and Handling Editor declared their shared affiliation, and the Handling Editor states that the process nevertheless met the standards of a fair and objective review.

## Supplementary material

Supplementary Figure S1 **Overview of *Gnetum parvifolium* tanscriptome sequencing and assembly. (A)** Classification of raw reads after filtering and trimming adapters; **(B)** Transcript and Unigene length interval, the x-axis represents the length interval; **(C/D)** Transcript/unigene length distribution, the y-axis represents the length frequency.

Supplementary Figure S2 **Candidate unigenes involved in phenylpropanoids biosynthesis in *Gnetum parvifolium***. Each enzyme name is followed by the number of unigenes homologous to gene families encoding this enzyme. *PAL*, phenylalanine ammonia-lyase; *C4H*, cinnamic acid 4-hydroxylase; *4CL*, 4-coumarate: CoA ligase; *HCT*, hydroxycinnamoyl-coenzyme A shikimate:quinate hydroxycinnamoyl-transferase; *C3*′*H*, p-coumaroyl shikimate 3′-hydroxylase; *CCoAOMT*, caffeoyl CoA 3-O-methyltransferase; *CCR*, cinnamoyl-CoA reductase; *F5H*, ferulate 5-hydroxylase; *COMT*, caffeic acid/5-hydroxyferulic acid O-methyltransferase; *CAD*, cinnamyl alcohol dehydrogenase; *HCALDH*, hydroxycinnamaldehyde dehydrogenase; *REF1*, coniferyl-aldehyde dehydrogenase; *PRX*, peroxidase; *PRD*, peroxiredoxin 6; *katG*, catalase-peroxidase; *UGT72E*, coniferyl-alcohol glucosyltransferase.

Supplementary Figure S3 **Candidate unigenes involved in flavonoids biosynthesis in *Gnetum parvifolium***. Each enzyme name is followed in parentheses by the number of unigenes homologous to gene families encoding this enzyme. The red number in the bracket following each gene name indicates the number of corresponding *G. parvifolium* unigenes. *C4H*, trans-cinnamate 4-monooxygenase; *CHS*, chalcone synthase; *CHI*, Chalcone Flavanone 3-hydroxylase; *F*3′5′*H*, flavonoid 3′,5′-hydroxylase; *FLS*, flavonol synthase; *F3H*, naringenin 3-dioxygenase; *F*3′*H*, flavonoid 3′-monooxygenase; *F3OMT*, flavonol 3-O-methyltransferase; *DFR*, flavanone 4-reductase; *LAR*, leucoanthocyanidin reductase; *ANR*, anthocyanidin reductase; *CCoAOMT*, caffeoyl CoA 3-O-methyltransferase.

Supplementary Figure S4 **Expression patterns of candidate gene *PAL-like* (comp69381_c0) involved in stilbenoids biosynthesis in different tissues from *Gnetum parvifolium* (A) and leaves of *Gnetum parvifolium* seedlings under high temperature/UV-C stresses (B/C)**. Detailed information as in Figures 6, 7.

Supplementary Figure S5 **Distribution of candidate SSRs motifs including repeats of mononucleotides involved in metabolism in *Gnetum parvifolium* tanscriptome**.

Supplementary Table S1 **Summary of *Gnetum parvifolium* Illumina transcriptome sequencing and function annotations**.

Supplementary Table S2 **Read counts and FPKM values of *Gnetum parvifolium* unigenes**.

Supplementary Table S3 **BLAST results against the NCBI NR database for all the unigenes**.

Supplementary Table S4 **Functional annotation of unigenes based on gene ontology (GO) classification. (A)** Biological process; **(B)** Cellular component; **(D)** Molecular function; **(D)** Total GO terms. In Figure 2, “Immune system” and “Locomotion” were excluded from Biological process; “Synapse” was excluded from Cellular component.

Supplementary Table S5 **Functional annotation of unigenes based on KOG classification. (A)** Main unigenes of KOG classification; **(B)** Unigenes of KOG subcategories; **(C)** Unigenes involved in secondary metabolism.

Supplementary Table S6 **Functional annotation of unigenes based on KEGG classification. (A)** Unigenes involved in metabolism; **(B)** Unigenes involved in secondary metabolites; **(C)** Total KEGG classification without removing pathways. In Figure 4, several pathways were excluded from the total including “Nervous system”, “Immune system”, “Digestive system”, “Endocrine system”, and “Circulatory system”.

Supplementary Table S7 **Identities of candidate genes involved in KEEG pathways of phenylpropanoids, flavonoids, and stilbenoids**. Unigenes with more than 40% identities with functionally validated enzymes were selected in Table 1. Unigenes of hit length less than 100 bp were marked with red color.

Supplementary Table S8 **Primers of candidate genes for qRT-PCR**. Primers marked with red color allowed successful amplification of four selected candidates in Figures 6, 7; primer marked with blue color allowed successful amplification showed in Supplementary Figure S4; other primers were not successfully amplified in this study.

Supplementary Table S9 **Simple sequence repeats (SSRs) in genes involved in secondary metabolism**.

Supplementary Table S10 **Summary of simple sequence repeats (SSRs) identified in transcripts of *Gnetum parvifolium***. Repeats of mononucleotides were excluded from the distribution of SSRs in different repeat types.

Supplementary Table S11 **Potential *STS-like* genes discovered in *Gnetum parvifolium* transcriptome**.
