# Supplementary figures and images for: Transcriptome Characterization of Gnetum parvifolium Reveals Candidate Genes Involved in Important Secondary Metabolic Pathways of Flavonoids and Stilbenoids
Source: Front Plant Sci. 2016 Mar 4;7:174. doi: 10.3389/fpls.2016.00174 (PMC4778121; doi:10.3389/fpls.2016.00174)

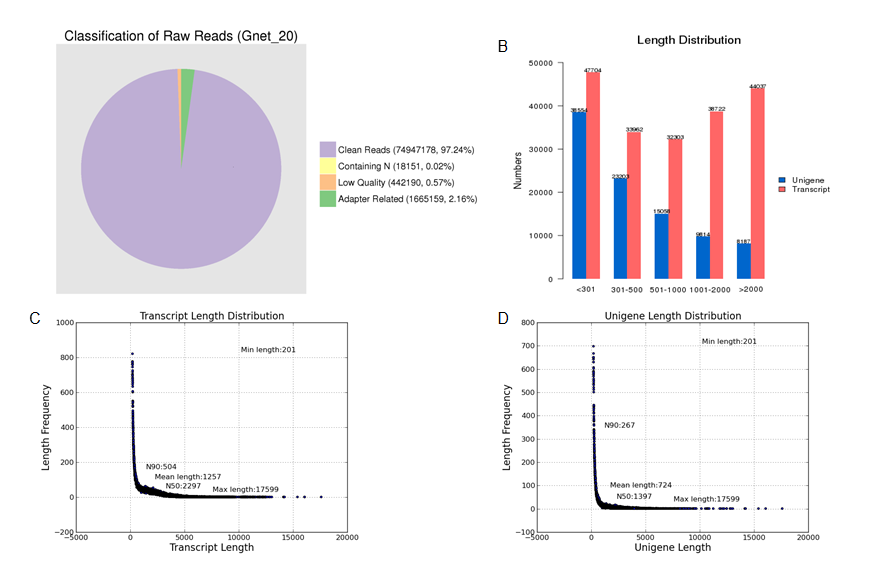

Supplement: Supplementary Figure S1 — Overview of Gnetum parvifolium tanscriptome sequencing and assembly. (A) Classification of raw reads after filtering and trimming adapters; (B) Transcript and Unigene length interval, the x-axis represents the length interval; (C/D) Transcript/unigene length distribution, the y-axis represents the length frequency. [file Image1.TIF]

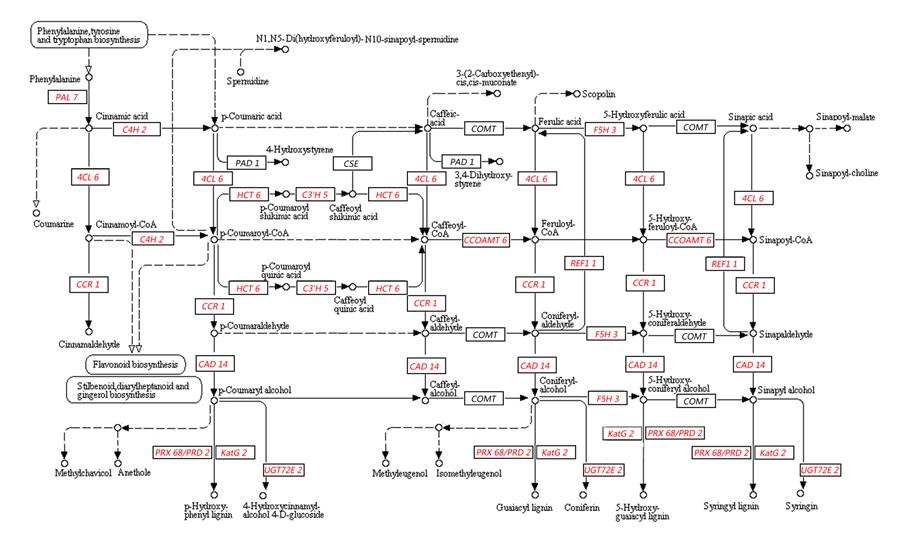

Supplement: Supplementary Figure S2 — Candidate unigenes involved in phenylpropanoids biosynthesis in Gnetum parvifolium. Each enzyme name is followed by the number of unigenes homologous to gene families encoding this enzyme. PAL, phenylalanine ammonia-lyase; C4H, cinnamic acid 4-hydroxylase; 4CL, 4-coumarate: CoA ligase; HCT, hydroxycinnamoyl-coenzyme A shikimate:quinate hydroxycinnamoyl-transferase; C3′H, p-coumaroyl shikimate 3′-hydroxylase; CCoAOMT, caffeoyl CoA 3-O-methyltransferase; CCR, cinnamoyl-CoA reductase; F5H, ferulate 5-hydroxylase; COMT, caffeic acid/5-hydroxyferulic acid O-methyltransferase; CAD, cinnamyl alcohol dehydrogenase; HCALDH, hydroxycinnamaldehyde dehydrogenase; REF1, coniferyl-aldehyde dehydrogenase; PRX, peroxidase; PRD, peroxiredoxin 6; katG, catalase-peroxidase; UGT72E, coniferyl-alcohol glucosyltransferase. [file Image2.TIF]

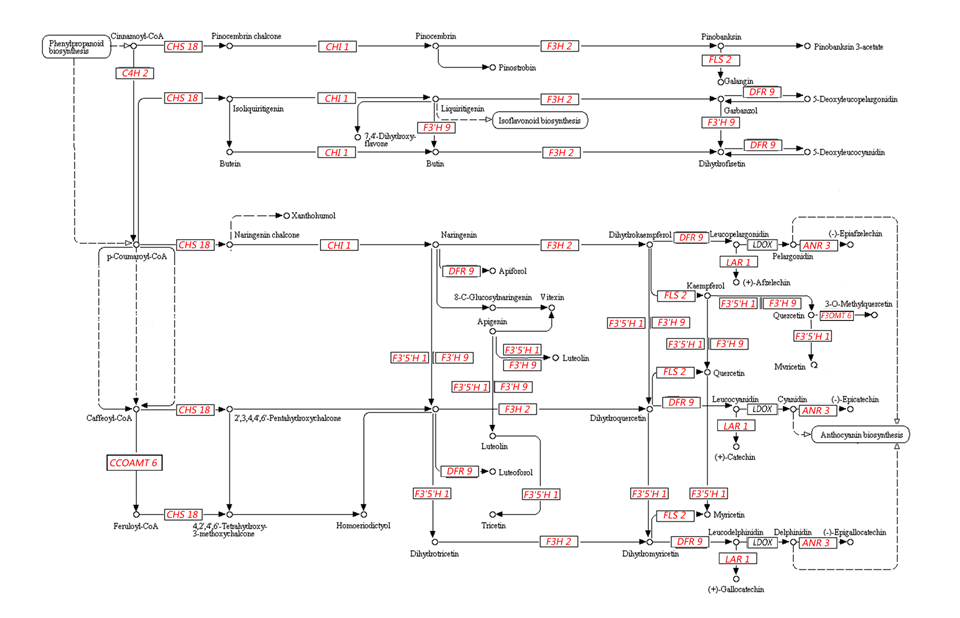

Supplement: Supplementary Figure S3 — Candidate unigenes involved in flavonoids biosynthesis in Gnetum parvifolium. Each enzyme name is followed in parentheses by the number of unigenes homologous to gene families encoding this enzyme. The red number in the bracket following each gene name indicates the number of corresponding G. parvifolium unigenes. C4H, trans-cinnamate 4-monooxygenase; CHS, chalcone synthase; CHI, Chalcone Flavanone 3-hydroxylase; F3′5′H, flavonoid 3′,5′-hydroxylase; FLS, flavonol synthase; F3H, naringenin 3-dioxygenase; F3′H, flavonoid 3′-monooxygenase; F3OMT, flavonol 3-O-methyltransferase; DFR, flavanone 4-reductase; LAR, leucoanthocyanidin reductase; ANR, anthocyanidin reductase; CCoAOMT, caffeoyl CoA 3-O-methyltransferase. [file Image3.TIF]

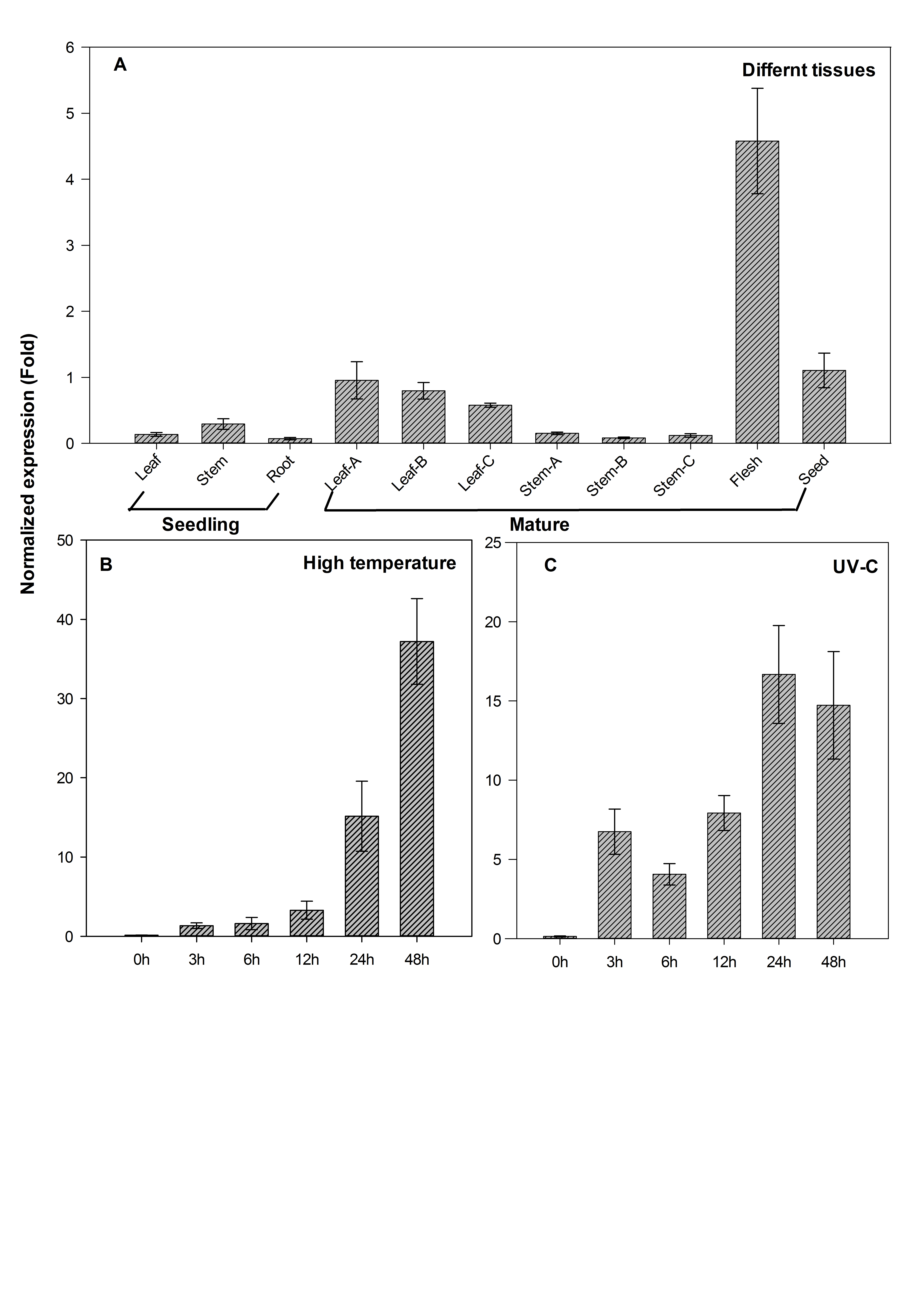

Supplement: Supplementary Figure S4 — Expression patterns of candidate gene PAL-like (comp69381_c0) involved in stilbenoids biosynthesis in different tissues from Gnetum parvifolium (A) and leaves of Gnetum parvifolium seedlings under high temperature/UV-C stresses (B/C). Detailed information as in Figures 6, 7. [file Image4.JPEG]

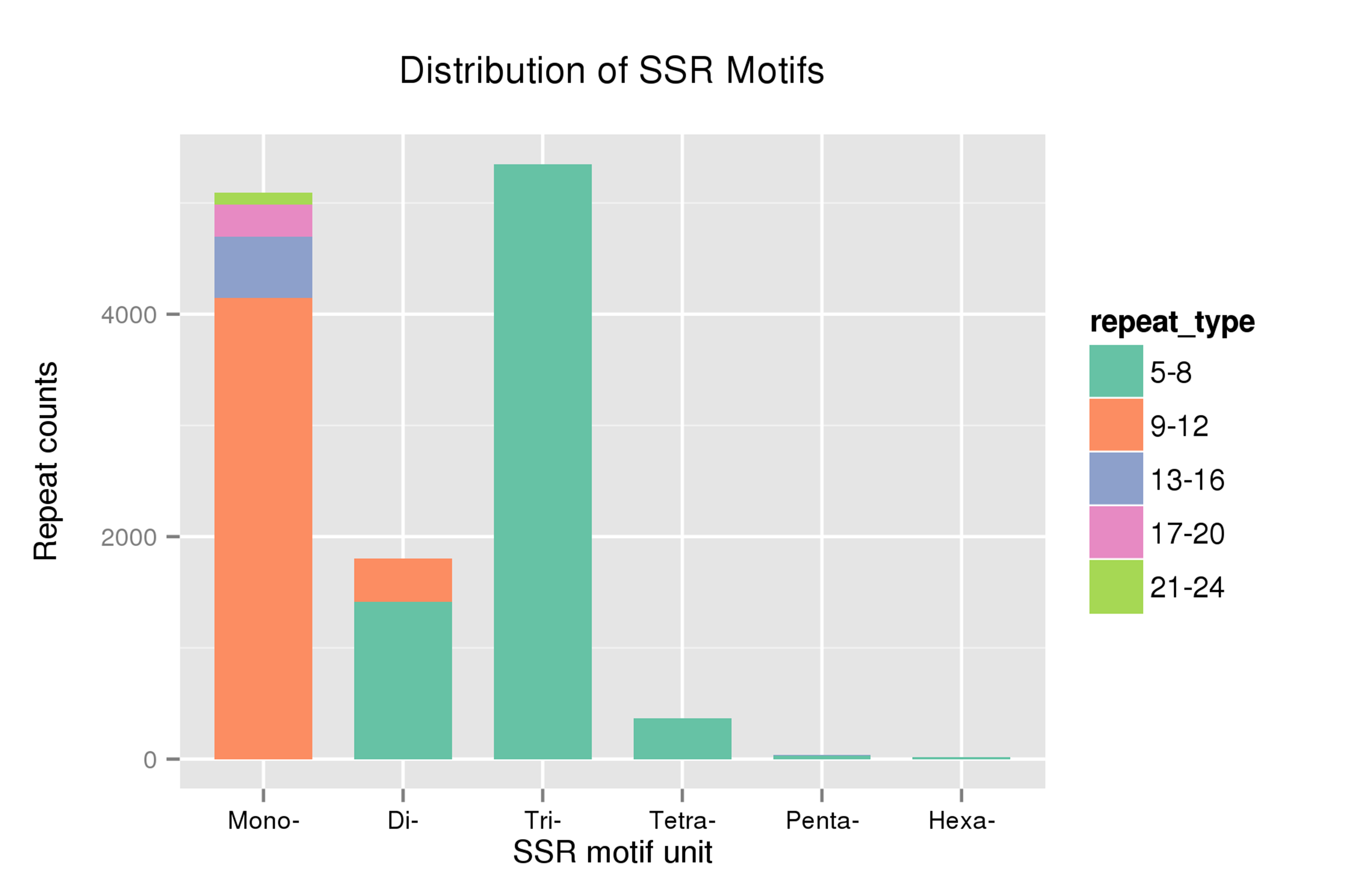

Supplement: Supplementary Figure S5 — Distribution of candidate SSRs motifs including repeats of mononucleotides involved in metabolism in Gnetum parvifolium tanscriptome. [file Image5.TIF]
