# Supplementary material for: Transcriptome Characterization of Gnetum parvifolium Reveals Candidate Genes Involved in Important Secondary Metabolic Pathways of Flavonoids and Stilbenoids
Source: Front Plant Sci. 2016 Mar 4;7:174. doi: 10.3389/fpls.2016.00174 (PMC4778121; doi:10.3389/fpls.2016.00174)
Supplement: Supplementary Table S1 — Summary of Gnetum parvifolium Illumina transcriptome sequencing and function annotations. [file Table1.DOC]

**Supplementary table S1A. Summary of transcriptome sequencing and assembly in *Gnetum parvifolium***

| Raw Reads | 77072678 |
| --- | --- |
| Clean Reads | 74947178 |
| Clean Bases | 9.37G |
| Error(%) | 0.03 |
| Q20(%) | 96.39 |
| Q30(%) | 92.61 |
| GC Content(%) | 44.95 |

**Supplementary table S1B. Summary of transcriptome sequencing for sequence information in *Gnetum parvifolium***

|  | Number | Min length | Mean length | Median length | Max length | N50 |
| --- | --- | --- | --- | --- | --- | --- |
| Transcripts | 196728 | 201 | 1257 | 709 | 17599 | 2297 |
| Unigenes | 94816 | 201 | 724 | 350 | 17599 | 1397 |

**Supplementary table S1C. Summary of transcriptomic functional annotation in *Gnetum parvifolium***.

|  | Number of unigenes | Percentage(%) |
| --- | --- | --- |
| Annotated in Nr | 21308 | 22.54 |
| Annotated in Nt | 5888 | 6.2 |
| Annotated in KO | 6627 | 6.98 |
| Annotated in SwissProt | 15359 | 16.19 |
| Annotated in PFAM | 20249 | 21.35 |
| Annotated in GO | 21498 | 22.67 |
| Annotated in KOG | 8643 | 9.11 |
| Annotated in all Databases | 2195 | 2.31 |
| Annotated in at least one Database | 27722 | 29.23 |
| Total Unigenes | 94816 | 100 |
