# Supplementary material for: Transcriptome Characterization of Gnetum parvifolium Reveals Candidate Genes Involved in Important Secondary Metabolic Pathways of Flavonoids and Stilbenoids
Source: Front Plant Sci. 2016 Mar 4;7:174. doi: 10.3389/fpls.2016.00174 (PMC4778121; doi:10.3389/fpls.2016.00174)
Supplement: Supplementary Table S8 — Primers of candidate genes for qRT-PCR. Primers marked with red color allowed successful amplification of four selected candidates in Figures 6, 7; primer marked with blue color allowed successful amplification showed in Supplementary Figure S4; other primers were not successfully amplified in this study. [file Table8.DOC]

**Supplementary talbe S8.**

| **Gene** | **Accession** | **Forward primer** | | **Reverse primer** |
| --- | --- | --- | --- | --- |
| *PAL-like* | comp84892_c0 | | GTGTACTTTGGGTAGTTGGTG | AATAACGGTCTTCGTGAGG |
|  | comp69381_c0 | | GGCTGACAAGTACGGAGGAGG | CGTGCAATGGTGGAAACG |
|  | comp62938_c0 | | GTCGGTGCCCTTGTTCATG | TCTGCCGTTTCTCAAATCTCG |
|  | comp55610_c0 | | AGTCTGGGTACGCCAAACCG | CGGAGGAGTGGCACAACAAGG |
|  | comp77470_c0 | | AACCATAACGGCGTCGGG | CGTTGGCGTCGAAGCACA |
|  | comp96780_c0 | | CGGCGTGACAACAAAGACC | GGGCGAGTACATTGAGAAGG |
|  | comp81110_c0 | | CCTTCAACTCCTCCTCGAACACGC | GCTTTCGTACATTGACAACC |
| *4CL-like* | comp94230_c0 | | TGCCAATCCCAACCTTCACCT | CCACCCTCACGGACGACAAATC |
|  | comp94627_c0 | | CTCCTCACCCATCCCTCCAT | ACTTCACCAGCCGCACCTTC |
|  | comp89267_c0 | | TATGGAACAGAGGCAGAACGC | TTGAGGAGGAGGAGGAAATCG |
|  | comp95498_c0 | | CAGTTTCTCGCCTGTCTCGG | AGGAGTAGTAGTGCGATGGAG |
|  | comp77367_c0 | | TTATCACTACCTCGGCTTGTCTG | GCTTATTGCATCAATGTCGCTCT |
|  | comp95407_c0 | | GTCATCCACGAAACCCACAT | CCCGTCCTTTCACTCAGTCCC |
| *C4H-like* | comp90938_c0 | | GGGGACGAAACCACGACCAG | GGAGACGACCTCAATCACAG |
|  | omp689299_c0 | | TGGATATGCAGACATGCCTC | ACTCACTTCTTACCACAAAT |
| *STS-like* | omp550004_c0 | | CAGCCTGCCAATGTGATAAAC | TTCAGCATCTCCTCCGTGAG |
|  | comp58900_c0 | | AGCGATTTCCCTTCCTTT | ATGCTGCTGTAGACCTTCCA |
|  | comp228100_c0 | | TACAAATGCCGACCAACG | CGAAGTGGCAACCGATGT |
| *CYP-like* | comp92132_c0 | | GGCGGTCCTGAGTCGTGTCGTT | GGCAATCCAGCAAGCACTCC |
|  | comp276514_c0 | | ATGCACCAGCACGATCCCTC | GCAGACTCGCCGTCCATTCC |
|  | comp46018_c0 | | ACAGGTGATATGTTAAAGCAGGAGGT | GCGTACCATGCCCATAGTGCC |
|  | comp60276_c0 | | CGGCGACGGTGACTGTCAATT | AGGAGGTGCTGTGCATGGTAAGG |
|  | comp85497_c0 | | GTCATCCGTTTCAGAGTTTCAGTTC | AGTCTTTACATGCGATCCTC |
|  | comp74684_c0 | | GTGGTGCTAAGACTGGGTGG | GAAAGTGCGAACGAGACGAG |
|  | comp90467_c0 | | GCTTCCCGTATTCTTCTTCC | TCGGTTATCAATCAAATCTG |
| *Actin* |  | | TTGTAGGTCGCCCTCGTC | CTCCCTGTTAGCCTTTGG |
