# Supplementary material for: Transcriptome Characterization of Gnetum parvifolium Reveals Candidate Genes Involved in Important Secondary Metabolic Pathways of Flavonoids and Stilbenoids
Source: Front Plant Sci. 2016 Mar 4;7:174. doi: 10.3389/fpls.2016.00174 (PMC4778121; doi:10.3389/fpls.2016.00174)
Supplement: Supplementary Table S10 — Summary of simple sequence repeats (SSRs) identified in transcripts of Gnetum parvifolium. Repeats of mononucleotides were excluded from the distribution of SSRs in different repeat types. [file Table10.DOC]

**Supplementary talbe S10.**

| **SSR mining** | **Number** |
| --- | --- |
| Total number of sequences examined | 94,816 |
| Total size of examined sequences (bp) | 68,693,366 |
| Total number of identified SSRs | 12,668 |
| Number of SSR containing sequences | 10,277 |
| Number of sequences containing more than one SSR | 1,818 |
| Number of SSRs present in compound formation | 633 |
| **Distribution of SSRs in different repeat types unit size** | **Number of SSRs** |
| Dinucleotide | 1802 (23.8%) |
| Trinucleotide | 5349 (70.6%) |
| Tetranucleotide | 368 (4.9%) |
| Pentanucleotide | 36 (0.5%) |
| Hexanucleotide | 17 (0.02%) |
| No. of total SSRs | 7572 |
